# Meetings of Medical and Dental Societies

**Published:** 1880-04

**Authors:** 


ALUMNI ASSOCIATION OF THE UNIVERSITY OF MARYLAND.—The annual meeting of the association was held at the Rennert House on the evening of March 5, 1880.

The following officers were elected for the ensuing year: President, Dr. G. W. Miltenberger; Vice-Presidents, Dr. Jas. Carey Thomas, Dr. Richard McSherry, Dr. D. I. McKew; Recording Secretary, Dr. Eugene F. Cordell; Corresponding Secretary, Dr. B. B. Browne; Treasurer, Dr. S. C. Chew. Dr. Miltenberger, on taking the chair, made a felicitous address, and proposed the following as the motto of the association: "Filius sim dignus istâ dignâ parente." Dr. Tiffany presented the graduating class of 1880. A committee was appointed to draft a constitution and by-laws. Two annual prizes of $100 and $50 respectively were established, to be conferred upon the two graduates passing the most satisfactory examinations at the university. Dr. Charles H. Cockey announced an annual prize of a case of amputating instruments, to be given to the graduate in the university who shall stand the best examination in surgery. The association then adjourned to partake of a supper provided by the faculty. The next meeting will be held May 1, 1880, to receive the report of the committee on constitution and by-laws.

THE following announcement has just been received, viz: The sixteenth annual meeting of the Illinois State Dental Society will be held at Bloomington on Tuesday, May 11th, and continue four days. Dentists from other States are cordially invited to be present.

EDMUND NOYES, Secretary.

The "Kansas State Dental Association" will hold its regular annual meeting in Ruggles and Plumb's Hall, Emporia, Kansas, commencing Tuesday, May 4th, 1880.

Those in attendance will be entertained at reduced rates ($1.50 per day,) at Merchants' and Park Place Hotels.

Members are especially requested to be present. A cordial invitation is extended to the profession throughout the State to meet and connect themselves with the Association.

Prof. James E. Garretson, M. D., D. D. S., has been made dean of the Philadelphia Dental College. We are confident that with his world-wide reputation, energy and management, the college will be prosperous and the course thorough.

Meetings of State Medical Societies, May, 1880:

Kentucky State Medical Society, at Lexington, May 4th.
Kansas State Medical Society, at Leavenworth, May 11th.
North Carolina Medical Society, in Wilmington, May 11th.
Michigan State Medical Society, at Grand Rapids, May 12th.
Illinois State Medical Society, at Belleville, May 18th.
Indiana State Medical Society, May 18th.
Missouri State Medical Society, at Carthage, May 18th.
Pennsylvania State Medical Society, at Altoona, May 19th.
New Jersey State Medical Society, at Princeton, May 25th.
The above information received just as we go to press.

